# Supplementary material for: Chromothripsis during telomere crisis is independent of NHEJ, and consistent with a replicative origin
Source: Genome Res. 2019 May;29(5):737–49. doi: 10.1101/gr.240705.118 (PMC6499312; doi:10.1101/gr.240705.118)
Supplement: Supplemental Material [file supp_gr.240705.118_Supplemental_file_1.zip › contigs/annotated_contigs/DB57/contig.2.DB57_length_327_mean_cov_4.12844036697.docx]

**DB57_length_327_mean_cov_4.12844036697**

```
CTGGCCTCTCAGTTTGATTACTGGGGCCATAAACAGCTTTCCCGTGAGAGCTGAGGTGAACATTTCTTGCTCATTAGGCCTCTTCCAGA
 >chr6:161942780-161942933 + E=9e-81
CAAGCCTCCCTTGCATGCAATGAATATGCACTTGATGCCCAGTGAACATGCACTTGATACTCAA|ACATGGTGAGGCTGG|GACTCACC
                                                                                >chr6:1
ATGCCCAGCCTCAGTGTAGAGTCTTTTCGAAGGCCTGGTTTCTGTTTAGCCCTTAGGGAAGGAAGCCTAGAGGTGGTTAGTGAGGGTGG
62389352-162389511 + E=2e-81
GGACATGACAGGACTGTCTGACCTCCCACCCCATCATGGCTGGAACTCAGCTTCCAAGGTGT
```
